# Cardiorespiratory fitness and morbidity and mortality in patients with non-small cell lung cancer: a prospective study with propensity score weighting

**DOI:** 10.1080/07853890.2023.2295981

**Published:** 2023-12-21

**Authors:** Yaoshan Dun, Ni Cui, Shaoping Wu, Siqian Fu, Jeffrey W. Ripley-Gonzalez, Nanjiang Zhou, Tanghao Zeng, Dezhao Li, Mi Chen, Yu Ren, Wan Yee Lau, Yang Du, Randal J. Thomas, Ray W. Squires, Thomas P. Olson, Suixin Liu

**Affiliations:** a Division of Cardiac Rehabilitation, Department of Physical Medicine and Rehabilitation, Xiangya Hospital of Central South University, Changsha, Hunan, China; b Division of Preventive Cardiology, Department of Cardiovascular Medicine, Mayo Clinic, Rochester, MN, USA; c National Clinical Research Center for Geriatric Disorders, Xiangya Hospital of Central South University, Changsha, Hunan, China; d School of Cardiovascular and Metabolic Medicine and Sciences, Faculty of Life Sciences and Medicine, King’s College London, United Kingdom; e Faculty of Medicine, The Chinese University of Hong Kong, Prince of Wales Hospital, Hong Kong, SAR, China; f Department of Neurology, Xiangya Hospital of Central South University, Changsha, Hunan, China

**Keywords:** Cardiopulmonary exercise test, cardiorespiratory fitness, perioperative morbidity, mortality, lung cancer, surgery

## Abstract

**Introduction:**

This study aimed to investigate the association between cardiorespiratory fitness (CRF) and perioperative morbidity and long-term mortality in operable patients with early-stage non-small cell lung cancer (NSCLC).

**Patients and Methods:**

This prospective study included consecutive patients with early-stage NSCLC who underwent presurgical cardiopulmonary exercise testing between November 2014 and December 2019 (registration number: ChiCTR2100048120). Logistic and Cox proportional hazards regression were applied to evaluate the correlation between CRF and perioperative complications and long-term mortality, respectively. Propensity score overlap weighting was used to adjust for the covariates. We performed sensitivity analyses to determine the stability of our results.

**Results:**

A total of 895 patients were followed for a median of 40 months [interquartile range 25]. The median age of the patients was 59 years [range 26–83], and 62.5% were male. During the study period, 156 perioperative complications and 146 deaths were observed. Low CRF was associated with a higher risk of death (62.9 versus 33.6 per 1000 person-years; weighted incidence rate difference, 29.34 [95% CI, 0.32 to 58.36] per 1000 person-years) and perioperative morbidity (241.6 versus 141.9 per 1000 surgeries; weighted incidence rate difference, 99.72 [95% CI, 34.75 to 164.70] per 1000 surgeries). A CRF of ≤ 20 ml/kg/min was significantly associated with a high risk of long-term mortality (weighted hazard ratio, 1.98 [95% CI, 1.31 to 2.98], *p* < 0.001) and perioperative morbidity (weighted odds ratio, 1.93 [1.28 to 2.90], *p* = 0.002) compared to higher CRF.

**Conclusion:**

The study found that low CRF is significantly associated with increased perioperative morbidity and long-term mortality in operable patients with early-stage NSCLC.

## KEY MESSAGES

1. Low cardiorespiratory fitness is significantly associated with increased perioperative morbidity and long-term mortality in operable patients with early-stage non-small cell lung cancer.
2. Future research is recommended to investigate the potential prognostic role of integrating cardiorespiratory fitness into the currently used prognosis algorithm for patients with non-small cell lung cancer.

## Introduction

Lung cancer is responsible for the highest cancer-related mortality worldwide, with five-year survival rates ranging from 26% to 64% [1]. Surgical lung resection remains the standard treatment for early-stage non-small cell lung cancer (NSCLC), comprising of approximately 80–85% of lung cancer cases [2]. This procedure, however, carries a relatively high risk of perioperative morbidity. The incidence rate is estimated 20% to 40% [3]. Therefore, investigating non-invasive methods for identifying surgical candidates who are at a high risk of perioperative morbidity and worse long-term prognosis has great clinical significance.

Cardiorespiratory fitness (CRF) serves as an indicator of comprehensive physiological cardiorespiratory function [4], and it finds broad applications across various clinical contexts [5,6]. The CRF directly measured by cardiopulmonary exercise testing (CPET), peak oxygen consumption ($\dot{V}$ O_2_peak), is recommended as a clinical vital sign by the American Heart Association [7]. Despite the reported association between CRF and perioperative morbidity in several surgical procedures [8], existing research linking CRF and perioperative morbidity in patients with NSCLC remains somewhat inconclusive, largely due to limited sample sizes in these studies [9–13]. As a result, the use and interpretation of CRF for presurgical assessment in lung resection candidates remain debatable.

Although CRF has emerged as a robust predictor of all-cause cardiovascular disease and cancer mortality in apparently healthy adults over the last three decades [14], only three studies with relatively small sample sizes have explored the relationship between CRF and mortality in patients with lung cancer, yielding inconclusive results [15–17]. Two studies suggest that lower CRF could correspond to an increased risk of all-cause mortality [15,17], while one study found no significant association [16]. Given these discrepancies, large-scale studies are necessary to further investigate the correlation between CRF and long-term all-cause mortality in patients with lung cancer. The results of such studies could provide clinicians with research evidence to help them determine whether CRF should be used to improve the management of patients with lung cancer.

This study aimed to investigate the association between directly measured CRF and perioperative morbidity and long-term mortality in operable patients with early-stage NSCLC using a relatively large sample size.

## Methods

### Study design and participants

**Figure 1.**
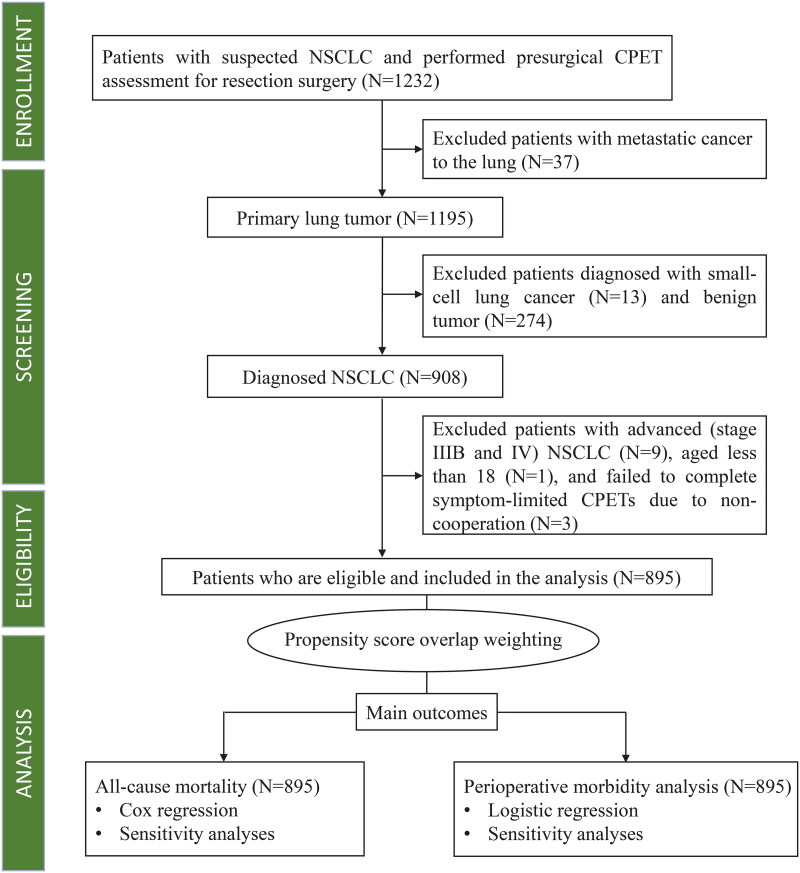
Flowchart from enrollment to analysis. CPET, cardiopulmonary exercise test; NSCLC, non-small cell lung cancer.

This prospective, observational study is part of the Xiangya Hospital Exercise Testing (X-ET) project [18,19]. Between November 1, 2014, and December 31, 2019, all suspected patients with NSCLC who underwent CPET one week before surgery were consecutively enrolled. The Multidisciplinary Tumour Board confirmed that the indications and contraindications for lung tumour resection surgery were consistent with current clinical practice guidelines [20,21]. We excluded patients with metastatic, advanced, and small-cell lung cancers, as well as benign tumours. Additionally, patients who were younger than 18 years or failed to complete a symptom-limited CPET were also excluded. A flowchart of patient inclusion and exclusion criteria is presented in Figure 1. The Ethics Committee of the Xiangya Hospital Central South University approved this study (approval number 202010145). Because this was an observational study with no impact on patient management, the requirement for informed consent was waived. The study was registered in the Chinese Clinical Trial Registry (registry number: ChiCTR2100048120) and reported following the guideline of the Strengthening the Reporting of Observational Studies in Epidemiology.

### Definition of exposed group

The $\dot{V}$ O_2_peak, which is the gold standard measure of CRF, was determined by symptom-limited CPET using a cycle ergometer with a ramp protocol (CARDIOVIT system [Schiller Switzerland]). All CPETs were performed following the standard exercise testing procedures [18,19], which were adapted from the Exercise Standards for Testing and Training published by the American Heart Association [22]. The optimal cut-off point of CRF for the outcomes was determined by the Receiver Operating Characteristics (ROC) curve and the Youden index [23]. Patients with CRF values equal to or less than the cut-off points were assigned to the exposed group.

### Perioperative management

All surgeries were performed by board-certified thoracic surgeons. The patients were managed by the same team of anaesthesiologists and surgeons. Following the surgery, patients underwent extubation in the surgical suite and managed in the post-anaesthesia recovery room for 24h. The patients were then transferred to the Department of Thoracic Surgery. Postoperative management was standardized, emphasizing early feeding, careful fluid balance, active mobilization, lung expansion exercises, and multimodal analgesia.

### Outcomes

#### Primary outcome

The primary outcome of this study was all-cause mortality after lung surgery, and before the study was censored on December 31, 2021. We confirmed mortality during the follow-up period using three independent methods: (1) the residents’ registration office, (2) the electronic medical record system, and (3) contacting participants’ families.

#### Secondary outcomes

The secondary outcomes of the study were complications that occurred during hospitalization and within 30 days of discharge. These complications included respiratory, cardiovascular, technical, and a composite of all complications. Respiratory complications include atelectasis, respiratory failure, pneumonia, pulmonary embolism, and acute respiratory distress syndrome. Cardiovascular complications include cardiac arrhythmias requiring drug therapy, acute coronary syndrome, cardiac failure, and stroke. Technical complications include chylothorax, prolonged lung air leakage, blood loss and massive haemothorax requiring blood transfusion, and wound or chest infections.

#### Covariates

At enrollment, we collected data on biological sex, age, smoking history, body mass index (BMI), medical history, and CPET parameters. Data on tumour histology, clinical stage, and type of lung resection were obtained after surgery using the electronic medical record system. We cross-checked all data for accuracy and anonymized patient information to maintain confidentiality.

#### Sample size

Typically, to ensure model accuracy, a minimum of ten equivalent deaths or perioperative complications is recommended for each adjusted covariate [24]. However, we used the propensity score overlap weighting technique to adjust covariates, which involves weighting and combining all covariates into one regression covariate. Thus, the 146 deaths and 156 complications observed were adequate for developing regression models.

### Statistical analysis

We conducted the Shapiro–Wilk test to evaluate the normality of continuous variables. Mean ± standard deviation was presented for normally distributed continuous variables while median (IQR) was presented for non-normally distributed variables. Categorical variables are reported as counts (percentages). Standardized mean difference (SMD) was used to measure the balance among individual covariates before and after propensity score weighting. Conventionally, an SMD less than 0.1 is deemed appropriate for balance [25].

To balance the covariates and minimize the impact of extreme propensities, we used the overlap weighting method to establish a propensity score model with covariates [26]. This method assigns weights based on the probability of an individual belonging to an alternate group [26,27].

The Kaplan-Meier method was used for time-to-event analyses and compared with the two-sided log-rank test. Cox proportional hazard models were fitted with weights derived from overlap weighting to assess the relationship between CRF and all-cause mortality. Additionally, crude and weighted incidence rates were computed as the mortality rate/1000 person-years [27].

We used a logistic regression model with propensity score overlap weighting to investigate the relationship between CRF and perioperative morbidity. We estimated the crude and weighted odds ratios as well as the perioperative morbidity rate (per 1000 surgeries).

Bootstrapping was used to assess the overall performance of the regression models [28,29]. We generated 1000 bootstrap samples to estimate the C-index, calibration, and Brier scores. Brier scores were evaluated on a scale of 0 (perfect accuracy) to 1 (perfect inaccuracy) to assess overall performance [30]. The calibration slope was used to assess consistency between observed and predicted hazards, with values close to 1 indicating good overall agreement [30]. Discrimination abilities were assessed with the C-index, where values of 0.5 and 1 indicated no discrimination and the best discrimination, respectively [30].

We conducted interaction tests for all measured confounders and used the E-value to evaluate the unmeasured confounders. The E-value is a statistical measure that determines the minimum association strength required for an unmeasured confounder to explain the observed relationship between exposure and outcome [31]. We also conducted pre-specified subgroup analyses based on birth sex, age, BMI, and smoking history. Before subgroup model development, overlap weighting propensity scores were recreated. Due to the potential for type I errors resulting from multiple comparisons, subgroup analyses were considered exploratory.

All analyses were performed using the R (version 4.2.0) software. The pROC, PSweight, tableone, cobalt, survival, survminer, fmsb, rms, boot, and E-value packages were used. Statistical significance was set at *p* < 0.05 (two-sided). The code and appropriate references for the statistical analyses are publicly available on the GitHub repository at https://github.com/YSDun/CRF/blob/3166c9378b911dc8c99ffe66f9b9c856c941b1b9/Code.Rmd.

## Results

### Participant characteristics

A total of 1232 patients suspected of having NSCLC underwent presurgical CPETs between November 1, 2014 and December 31, 2019. After the screening process, 895 patients were eligible and included in the study, while 337 were excluded because of metastatic lung cancer (*n* = 37), small cell lung cancer (*n* = 13), benign tumours (*n* = 274), stage IIIB and IV cancer (*n* = 9), failure to complete symptom-limited CPETs (*n* = 3), and age ≤ 18 years (*n* = 1) (Figure 1). The median age of the 895 participants was 59 years (IQR, 13 years), and the majority of the participants were males (62.5%). The characteristics of the CPET data are shown in Supplement eTable 1. The ROC analysis and Youden index identified CRF ≤20 ml/kg/min as an optimal cut-off value for prognosis in this study, as illustrated by the ROC curves in Supplement eFigure 2. Among the 895 participants, 234 (26.1%) had a CRF ≤20 ml/kg/min, while 661 (73.9%) had a CRF >20 ml/kg/min. The mean CRF was 22.9 ml/kg/min (SD, 4.5).

Females, older people, those with higher BMI, and those with a history of hypertension, diabetes mellitus, and coronary artery disease were more likely to have low CRF. Further details are presented in Table 1. The Love plot displaying the covariates balance before and after weighting is provided in Supplement eFigure 1.

**Table 1.** Demographic and clinical characteristics grouped by high and low CRF, before and after applying propensity score overlap weighting.

	Crude			Weighted^a^		
Variable	CRF≤ 20 mL/kg/min (*n* = 234)	CRF> 20 mL/kg/min (*n* = 661)	Standardized mean difference	CRF≤ 20 mL/kg/min	CRF> 20 mL/kg/min	Standardized mean difference
Male,	105 (44.9)	454 (68.7)	0.50	53.0	53.0	<0.001
Age, mean (SD), years	63 ± 9	57 ± 9	0.71	61 ± 9	61 ± 7	<0.001
Body mass index, mean (SD), kg/m^2^	24.1 ± 3.1	23.4 ± 3.2	0.23	23.9 ± 3.1	23.9 ± 3.9	<0.001
Smoking ever,	80 (34.2)	320 (48.4)	0.29	39.4	39.4	<0.001
Medical history,						
Hypertension	80 (34.2)	113 (17.1)	0.40	27.9	27.9	<0.001
Dyslipidaemia	19 (8.1)	70 (10.6)	0.09	8.6	8.6	<0.001
Diabetes mellitus	28 (12.0)	49 (7.4)	0.15	11.7	11.7	<0.001
Coronary artery disease	65 (27.8)	109 (16.5)	0.27	23.4	23.4	<0.001
Type of lung resections,			0.29			<0.001
Pneumonectomy	6 (2.6)	28 (4.2)		3.2	3.2	
Lobectomy	216 (92.3)	589 (89.1)		92.7	92.7	
Segmentectomy	6 (2.6)	10 (1.5)		1.8	1.8	
Wedge resection	4 (1.7)	3 (0.5)		0.8	0.8	
Two lung lobes	2 (0.9)	25 (3.8)		1.3	1.3	
Histology,			0.26			<0.001
Adenocarcinoma	186 (79.5)	452 (68.4)		75.3	75.3	
Squamous cell	40 (17.1)	171 (25.9)		20.9	20.9	
Other NSCLCs	8 (3.4)	38 (5.7)		3.8	3.8	
Clinical Stage,			0.28			<0.001
0	7 (3.0)	12 (1.8)		2.1	2.1	
I	166 (70.9)	392 (59.3)		68.7	68.7	
II	24 (10.3)	97 (14.7)		11.9	11.9	
IIIA	37 (15.8)	160 (24.2)		17.4	17.4	

CRF: cardiorespiratory fitness; NSCLC: non-small cell lung cancer; SD: standard deviation. ^a^After applying overlap weighting, a single individual no longer corresponds to a single data entity. Consequently, raw counts are no longer not reported for categorical variables, and instead, the percentages are presented.

### The association between CRF and all-cause mortality

**Figure 2.**
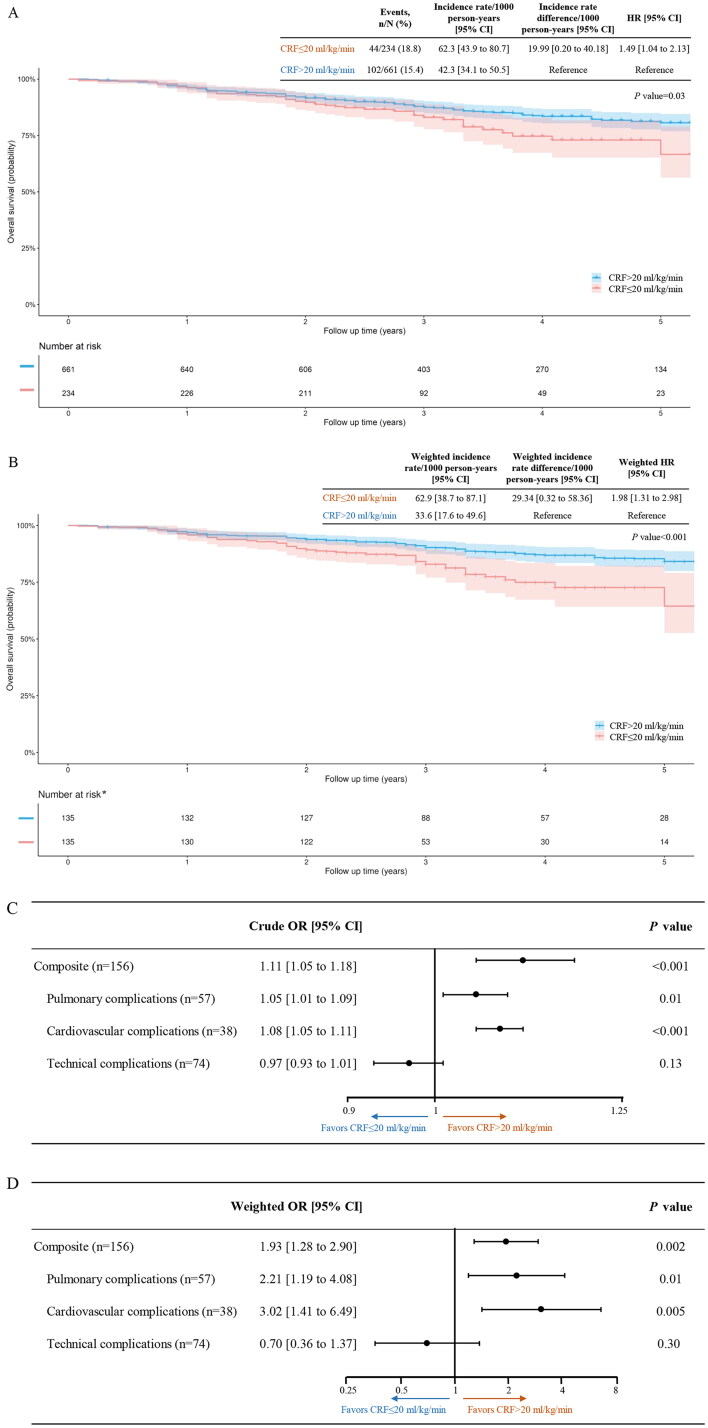
Kaplan–Meier survival curves (A–B) and Forest plots (C–D), before and after applying propensity score overlap weighting (*N* = 895). CRF, cardiorespiratory fitness; HR, hazard ratio; or, odds ratio. The shadow, along with the curves, represents the 95% confidence interval. *after propensity score overlap weighting, a single individual no longer represents a single data entity.

The median follow-up period was 40 months (IQR, 25 months). A total of 146 patients died during the study. After applying propensity score overlap weighting, the death rates per 1000 person-years were 62.9 in the low and 33.6 in the high CRF groups, respectively. The weighted incidence rate difference (IRD) between the two groups was 29.34 [95% CI, 0.32 to 58.36] per 1000 person-years. Participants with a CRF of ≤20 ml/kg/min had a 1.98 times higher risk of death than those with a CRF of >20 ml/kg/min  (weighted HR, 1.98 [95% CI, 1.31 to 2.98]) (Figure 2).

**Figure 3.**
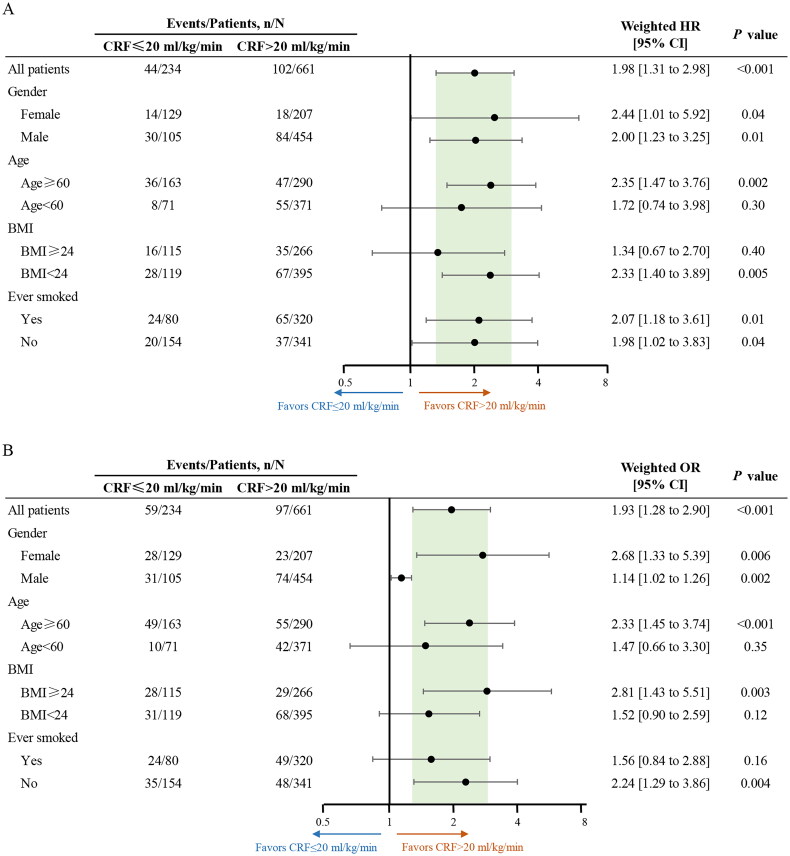
Subgroup analyses on the association between measured CRF and all-cause mortality (A) and perioperative morbidity (B). BMI, body mass index; CRF, cardiorespiratory fitness; HR, hazard ratio; or, odds ratio.

Several sensitivity analyses were conducted to evaluate the potential sources of bias that might impact the observed association between CRF and all-cause mortality. First, interaction tests were conducted to examine the role of the measured covariates. When testing for interactions, only age exhibited a significant interaction with the observed association between CRF and all-cause mortality (Supplement eTable 2). Pre-specified subgroup analyses were also conducted, revealing that the relationship between CRF and all-cause mortality remained statistically significant across the male, female, age ≥ 60 years, BMI < 24 kg/m^2^, ever smoked, and never smoked subgroups (Figure 3).

Moreover, this study employed the E-value test to evaluate the potential for bias resulting from unmeasured confounders. The calculated E-value was 2.58, which exceeds the HRs of most recognized risk factors, such as male sex (HR = 1.29), for lung cancer prognosis as reported in previous literature [32], indicating that the observed association between CRF and all-cause mortality is less likely to be explained by an unmeasured confounder.

In addition, we conducted internal validation using bootstrapping resampling to assess the robustness of the observed association. The analyses showed that the Brier score was 0.11 [95% CI, 0.10 to 0.12], the calibration slope was 0.85 [0.81 to 0.89], and the C-index was 0.62 [0.51 to 0.73]. These results indicate good overall performance of the regression model on the relationship between CRF and all-cause mortality [30].

### The association between CRF and perioperative morbidity

Among the 895 participants, 156 experienced a total of 169 perioperative complications, including 57 pulmonary complications, 38 cardiovascular complications, and 74 technical-related complications. There were 241.6 and 141.9 complications per 1000 surgeries among patients with low and high CRF, respectively (weighted IRD per 1000 surgeries, 99.72 [95% CI, 34.75 to 164.70]). Participants with a CRF of ≤20 ml/kg/min had a 1.93 times higher risk of perioperative complications than those with a CRF of >20 ml/kg/min, with a weighted OR of 1.93 [95% CI, 1.28 to 2.90] (Figure 2). Table 2 presents the incidence rates and corresponding ORs for pulmonary, cardiovascular, and technical-related complications.

**Table 2.** Association of measured CRF with all-cause mortality and perioperative morbidity in operable patients with early-stage NSCLC, after applying propensity score overlap weighting.

Outcome	CRF≤ 20 mL/kg/min (*n* = 234)	CRF> 20 mL/kg/min (*n* = 661)	Weighted incidence rate difference/1000 person-years [95% CI]*^b^*	Weighted HR [95% CI]	
	Weighted incidence rate/1000 person-years				*P* value
**All-cause mortality** (n = 146)	62.9	33.6	29.34 [0.32 to 58.36]	1.98 [1.31 to 2.98]	<0.001
**Perioperative morbidity**	**Weighted incidence rate/1000 surgeries**		**Weighted difference/1000 surgeries (95% CI)**	**Weighted OR [95% CI]**	
Composite (n = 156)	241.6	141.9	99.72 [34.75 to 164.70]	1.93 [1.28 to 2.90]	0.002
Pulmonary complications (n = 57)	100.7	48.3	52.36 [8.04 to 96.69]	2.21 [1.19 to 4.08]	0.01
Cardiovascular complications (n = 38)	85.8	30.2	55.68 [16.37 to 94.98]	3.02 [1.41 to 6.49]	0.005
Technical complications (n = 74)	62.4	86.6	−24.26 [−67.35 to 18.82]	0.70 [0.36 to 1.37]	0.30

CRF: cardiorespiratory fitness; HR: hazard ratio; OR: odds ratio. For composite analysis. Only one event was included if the patient developed more than one perioperative complication.

Coronary artery diseases and clinical stage exhibited significant interactions with the observed association between CRF and perioperative morbidity (Supplement eTable 2). In the subgroup analyses, the association between CRF and perioperative morbidity remained statistically significant across the male, female, age ≥ 60 years, BMI ≥ 24 kg/m^2^, and never smoked groups (Figure 3). The calculated E-value was 2.12, which exceeded the HRs of the most recognized risk factors for perioperative morbidity in patients with NSCLC, indicating a lower likelihood of the observed association being explained by an unmeasured confounding variable. The results of internal validation analysis showed that the Brier score was 0.14 [95% CI, 0.13 to 0.16], the calibration slope was 0.78 [0.75 to 0.81], and the C-index value was 0.60 [0.53 to 0.67]. These findings indicate that the regression model for the association between CRF and perioperative morbidity demonstrated good overall performance [30].

## Discussion

This study investigated the association between CRF and perioperative morbidities and long-term mortality in a relatively large sample size of patients with early-stage NSCLC using propensity score overlap weighting. The results indicate a significant association between low CRF and both heightened perioperative morbidity and elevated long-term mortality rates in patients with early-stage NSCLC (eFigure 4).

While CRF is well-established as a robust predictor of cancer incidence [33], cancer-related mortality [34], and all-cause mortality in apparently healthy adults [32], studies assessing the relationship between CRF and all-cause mortality in patients with lung cancer are scarce, with inconsistent results. For example, Jones et al. demonstrated that CRF significantly predicted survival in a study with 398 patients with NSCLC [16], whereas Lindenmann et al. found no notable association between cancer-related death and CRF [17]. Notably, Cundrle et al. [35] demonstrated that CRF may not be the optimal predictor for CPET parameters related to cardiovascular complications in lung resection. This study, which features the largest sample size and longest follow-up period to date, supports the hypothesis that CRF is significantly associated with all-cause mortality in patients eligible for surgery with early-stage NSCLC. Further investigations are imperative to explore the prognostic implications of combining CRF with the other established CPET predictors [36] to improve outcomes in NSCLC patients.

In our study, 146 out of 895 NSCLC patients passed away during a median follow-up period of 40 months (IQR, 25 months). It is noteworthy that the mortality rate might be slightly elevated compared to the latest statistics. This variation could be attributed to diverse cancer stages, age and general health of patients, the effectiveness of the treatment plan, or advancements in medical treatment techniques over the years.

The role of CRF in pre-surgical assessment of patients with NSCLC remains a contentious issue. While certain studies indicate that CRF as a valuable marker for predicting perioperative morbidity, others present conflicting results. From a systematic literature search, six out of fourteen studies revealed a significant association between low CRF and a heightened risk of perioperative complications in lung resection [13,37–41], yet the remaining eight did not show any such correlation [9–12,42–45]. Moreover, there exists a lack of consensus on the optimal cut-off value for CRF in NSCLC patients. While 20 ml/kg/min is commonly utilized as the cut-off in heart failure patients [46], a study by Jones et al. [16] proposed different peak VO2 cut-offs, specifically ‘<13.9 ml/kg/min, 14.0–17.3 ml/kg/min, and >17.4 ml/kg/min’ in NSCLC patients. In our study involving Chinese NSCLC patients (median age, 59 years) undergoing lung resection, we observed that a CRF of ≤20 ml/kg/min was associated with a higher rate of perioperative morbidity. Two factors may contribute to this disparity. Firstly, Jones et al. determined the cut-off through tertile split of all patient distributions, while we established the cut-off *via* ROC analysis and the Youden index. Additionally, the difference may be influenced by ethnicities, as our prior research has highlighted variations in the normal CRF range across different ethnic groups [18,47]. A future multicentre study that includes participants from multiple countries is warranted to determine an optimal CRF cut-off point for identifying patients with NSCLC at an increased risk of perioperative morbidity.

Although CRF has been linked to patient prognosis across various health conditions, the underlying mechanisms remain elusive. A recent study suggested that the mitochondrial oxygen affinity of skeletal muscles may be closely associated with CRF [48]. Supporting this notion, our pre-clinical research demonstrated an association between mitochondrial function and the ability of mice to resist stress-induced myocardial [49] and skeletal muscle damage [50,51]. Thus, we conducted a pilot experiment to evaluate the correlation between all-cause mortality and the expression of a mitochondrial volume biomarker, 2-oxoglutarate dehydrogenase E1 component (OGDH), using immunohistochemical staining with an OGDH antibody. The results showed that OGDH expression in both tumour and tumour-adjacent tissues in the death group was significantly lower than that in the survival group (Supplementary eFigure 3). These findings suggest that mitochondrial function may be one of the mechanisms underlying the relationship between CRF and disease prognosis. Future studies are needed to verify this hypothesis and explore other mechanisms underlying the association between low CRF and adverse events.

**Figure 4.**
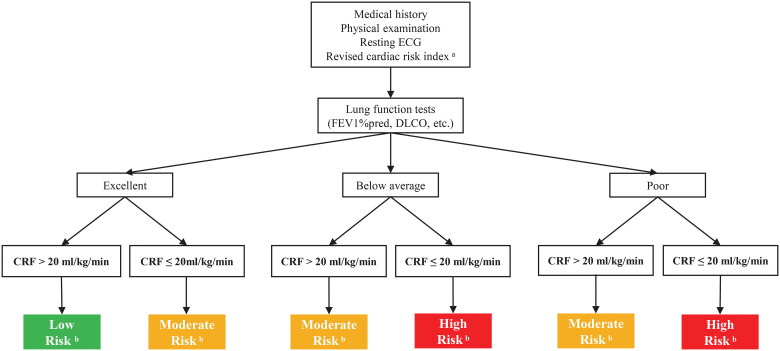
A Suggested algorithm for using CRF in the assessment of perioperative morbidity risk and long-term mortality in operable patients with early-stage NSCLC. CRF, cardiorespiratory fitness; DLCO, diffusing capacity of the lungs for carbon monoxide; ECG, electrocardiograph; FEV1, forced expiratory volume in one second. ^a^Revised cardiac risk index: (1) high-risk surgery (including lobectomy or pneumonectomy), (2) ischaemic heart disease (prior myocardial infarction, angina pectoris), (3) heart failure, (4) insulin-dependent diabetes, (5) previous stroke of transient ischemic attack, and (6) creatinine ≥2 mg·dL^−1^. ^b^Low risk in green indicates excellent prognosis and a low risk of perioperative complications. Moderate risk in yellow and high risk in red suggest a progressively worse prognosis and higher a risk of perioperative complications. Patients who are classified as moderate- and high-risk warrant strong consideration of more aggressive medical management and surgical options.

Clinical outcomes are widely acknowledged to be influenced by various factors in real-world settings. Previously established risk models, including The American College of Surgeons National Surgical Quality Improvement Program Surgical Risk Calculator [52] and The Society of Thoracic Surgeons Adult Cardiac Surgery Risk Model [53], has been validated their effectiveness in predicting risk. Aligning with current clinical guidelines for CRF usage in lung cancer patients, we propose an algorithm that incorporates CRF measurement as an additional parameter with a cut-off point of 20 ml/kg/min. This approach aims to assess perioperative morbidity risk and long-term prognosis in operable patients with early-stage NSCLC (Figure 4) [54,55]. Further investigations are essential to explore the prognostic significance of combining CRF measurement with other established and emerging predictors and risk models [52,53] to enhance outcomes in NSCLC patients.

### Limitations

This study has several limitations that should be considered. First, despite using the propensity score overlap weighting technique and conducting multiple sensitivity analyses, the possibility of residual and unmeasured confounding factors could not be fully excluded. Second, although we provided information regarding clinical staging, which can affect the eligibility and accessibility of neoadjuvant radiotherapy and chemoradiotherapy, we did not specifically address these treatments. Third, caution should be exercised when interpreting the subgroup results because the number of events in some subgroups was limited.

## Conclusions

Reduced CRF is significantly associated with perioperative morbidity and long-term all-cause mortality in operable patients with early-stage NSCLC. Future studies are recommended to investigate the potential prognostic role of integrating CRF into the currently used prognosis algorithm for patients with NSCLC eligible for surgery.

## Supplementary Material

Supplemental Material

## Data Availability

The data that support the findings of this study are available from the corresponding author, SXL, upon reasonable request.
