# Supplementary material for: Cardiorespiratory fitness and morbidity and mortality in patients with non-small cell lung cancer: a prospective study with propensity score weighting
Source: Ann Med. 2023 Dec 21;55(2):2295981. doi: 10.1080/07853890.2023.2295981 (PMC10763904; doi:10.1080/07853890.2023.2295981)
Supplement: Supplemental Material [file IANN_A_2295981_SM0554.docx]

**eTable 1.** Characteristics of Symptom-Limited Cardiopulmonary Exercise Testing.

|  | **Number or Mean** | **Percentage(%) or Standard Deviation** |
| --- | --- | --- |
| Cardiorespiratory fitness, ml/kg/min | 22.9 | 4.5 |
| Peak workload, watts | 102 | 29 |
| Peak HR, bpm | 148 | 16 |
| Peak systolic blood pressure peak, mmHg | 185 | 24 |
| Peak diastolic blood pressure peak, mmHg | 90 | 13 |
| Exhaustion/Good effort* | 874 | 98.20 |
| Achieved 85% of predicted maximal HR | 711 | 79.44 |
| RPE ≥17 | 761 | 85.03 |
| RER ≥1.05 | 598 | 66.82 |
| RER ≥1.10 | 424 | 47.37 |
| Presence of plateau of HR or oxygen consumption | 6 | 0.67 |
| Presence of clinical symptoms | 214 | 23.91 |
| Dizziness | 150 | 16.76 |
| Abnormal blood pressure response | 50 | 5.59 |
| Chest pain or distress | 22 | 2.46 |
| Skeletomuscular limit | 9 | 1.01 |
| ST segment elevation or depression ≥3 mm | 2 | 0.22 |

HR, heart rate; RPE, rating of perceived exertion; RER, respiratory exchange ratio.

* Participants were deemed to have been exhausted or put in a good effort during the cardiopulmonary exercise test if they met one of the following criteria: achieved 85% of predicted maximal HR; RPE as ≥17; RER was ≥1.05; demonstrated the presence of plateau of HR or oxygen consumption; or had clinical symptoms. (N=895)

**eTable 2.** Interaction *P* values for All-Cause Mortality and Perioperative Morbidity.

| **Factors** | **All-cause mortality** | **Perioperative morbidity** |
| --- | --- | --- |
| Sex | 0.26 | 0.29 |
| Age | 0.047 | 0.54 |
| BMI | 0.08 | 0.98 |
| Smoking ever | 0.39 | 0.47 |
| Hypertension | 0.10 | 0.87 |
| Dyslipidemia | 0.26 | 0.13 |
| Diabetes mellitus | 0.12 | 0.16 |
| Coronary artery diseases | 0.96 | 0.03 |
| Histology | 0.24 | 0.31 |
| Type of resection | 0.75 | 0.09 |
| Clinical stage | 0.96 | 0.001 |

BMI, body mass index. (N=895)

**eFigure 1.** The Love plot. BMI, body mass index; NSCLC, non-small cell lung cancer (N=895).

**eFigure 2.** Receiver operating characteristics curves (N=895).

**eFigure 3.** Immunohistochemical staining results of 2-oxoglutarate dehydrogenase E1 component protein in tumor and tumor-adjacent tissues. (a) Representative images of immunohistochemical staining for 2-oxoglutarate dehydrogenase E1 component (OGDH) protein in tumor and tumor-adjacent tissues between the death and survival groups. Brown color indicates positive staining. Four larger images were captured at 10 times magnification, and the other four in the corner were shot at 40 times magnification with the same view as those in dotted boxes; (b) Immunohistochemistry index (optical density * percentage of OGDH staining) among groups (death group-tumor tissue [N=20], death group-tumor adjacent tissue [N=20], survival group-tumor tissue [N=20], survival group-tumor adjacent tissue [N=20]).

As a pilot experiment, we detected the expression of a mitochondrial volume biomarker, OGDH, in lung tumor and tumor-adjacent tissues using immunohistochemistry with an OGDH antibody (15212-1-AP, Proteintech). The staining intensity (optical density [OD] of staining) multiplied by the percentage of staining (%Pos) indicated the amount of OGDH. The OD and %Pos were measured using digital image analysis. All available paired tumor and tumor-adjacent tissues from dead patients were assigned to the death group. Survivors who had clinical conditions similar to those of the death group were matched by propensity scores matching. We then assessed the difference in OD*%Pos of OGDH of lung tumor and tumor-adjacent tissues between the death and survival groups using the Wilcoxon rank sum test. OGDH expression in both tumor and tumor-adjacent tissues of the death group was significantly lower than that in the survival group (adjusted mean difference in OD*%Pos of OGDH, tumor tissue, 0.51 [95% CI, 0.11 to 0.90]; tumor-adjacent tissue, 0.59 [0.04 to 1.13]). Additionally, the amount of OGDH expressed in tumor-adjacent tissues was significantly higher than that in tumor tissue.

**eFigure 4.** Graphic abstract.
